# Integrated Genomics Identifies miR-32/MCL-1 Pathway as a Critical Driver of Melanomagenesis: Implications for miR-Replacement and Combination Therapy

**DOI:** 10.1371/journal.pone.0165102

**Published:** 2016-11-15

**Authors:** Prasun J. Mishra, Pravin J. Mishra, Glenn Merlino

**Affiliations:** 1 Department of Biochemical and Cellular Pharmacology, Genentech Inc., South San Francisco, California, United States of America; 2 Laboratory of Cancer Biology and Genetics, National Cancer Institute, National Institutes of Health, Bethesda, Maryland, United States of America; 3 Precision Genomics, Intermountain Healthcare, Dixie Regional Medical Center, St. George, Utah, United States of America; University of Connecticut Health Center, UNITED STATES

## Abstract

**Aims:**

Cutaneous malignant melanoma is among the deadliest human cancers, broadly resistant to most clinical therapies. A majority of patients with *BRAF*^V600E^ melanomas respond well to inhibitors such as vemurafenib, but all ultimately relapse. Moreover, there are no viable treatment options available for other non-*BRAF* melanoma subtypes in the clinic. A key to improving treatment options lies in a better understanding of mechanisms underlying melanoma progression, which are complex and heterogeneous.

**Methods:**

In this study we integrated gene and microRNA (miRNA) expression data from genetically engineered mouse models of highly and poorly malignant melanocytic tumors, as well as available human melanoma databases, and discovered an important role for a pathway centered on a tumor suppressor miRNA, *miR-32*.

**Results:**

Malignant tumors frequently exhibited poor expression of *miR-32*, whose targets include *NRAS*, *PI3K* and notably, *MCL-1*. Accordingly, *MCL-1* was often highly expressed in melanomas, and when knocked down diminished oncogenic potential. Forced *MCL-1* overexpression transformed immortalized primary mouse melanocytes, but only when also expressing activating mutations in *BRAF*, *CRAF or PI3K*. Importantly, both *miR-32* replacement therapy and the *MCL-1*-specific antagonist sabutoclax demonstrated single-agent efficacy, and acted synergistically in combination with vemurafenib in preclinical melanoma models.

**Conclusions:**

We here identify *miR-32*/*MCL-1* pathway members as key early genetic events driving melanoma progression, and suggest that their inhibition may be an effective anti-melanoma strategy irrespective of *NRAS*, *BRAF*, *and PTEN* status.

## Introduction

Cancer initiation and progression are stepwise processes, occurring over time via accumulation of mutations that activate oncogenes, deactivate pro-apoptotic and tumor suppressor genes, leading to tumorigenesis and, ultimately metastasis. This problem is magnified in malignant melanoma, which is highly metastatic and characteristically resistant to traditional therapies with a median patient survival of ~6 months. Recently developed targeted *BRAF*^V600E^ inhibitors (e.g. vemurafenib, dabrafenib) typically elicit significant clinical responses for most *BRAF*^*V600E*^ positive melanomas [1]. Unfortunately, melanoma patients routinely succumb to inherent and acquired resistance to targeted *BRAF*^*V600E*^ inhibitors [2], resulting in drug resistant progressive disease and an unacceptably high incidence of mortality [3].

Mutant RAS has thus far not been druggable, and agents that target downstream effectors in RAS/NF1-regulated networks (e.g. MEK inhibitors such as trametinib) have been only marginally effective as single agent therapies. Recently clinical success has been achieved using biological immunomodulatory therapies designed to enhance the effector arm of the immune system by targeting inhibitory mechanisms using antibody-based immune checkpoint inhibitors of CTLA-4, PD-1 and PD-L1 (e.g. ipilimumab; nivolumab, MK3475; BMS-936559, MEDI-4736, respectively). These immunomodulatory agents can produce durable clinical responses, but only 15%-30% of patients respond at all [4–6]. Meanwhile, there are no effective treatment options available for wildtype-*BRAF*/*NRAS* melanomas, which constitute ~30% of all melanomas. Melanomagenesis is significantly enhanced upon loss of melanoma-associated loci *PTEN* and *CDKN2A*. *CDKN2A* locus harbors two functionally distinct, yet overlapping tumor suppressors (*p16*^*INK4a*^ or INK4a, and *p14*^*ARF*^ or ARF), encoded in different reading frames. Moreover, the propensity of melanomas to metastasize, even many years after removal of the primary tumor, makes this cancer especially deadly.

**Fig 1:**
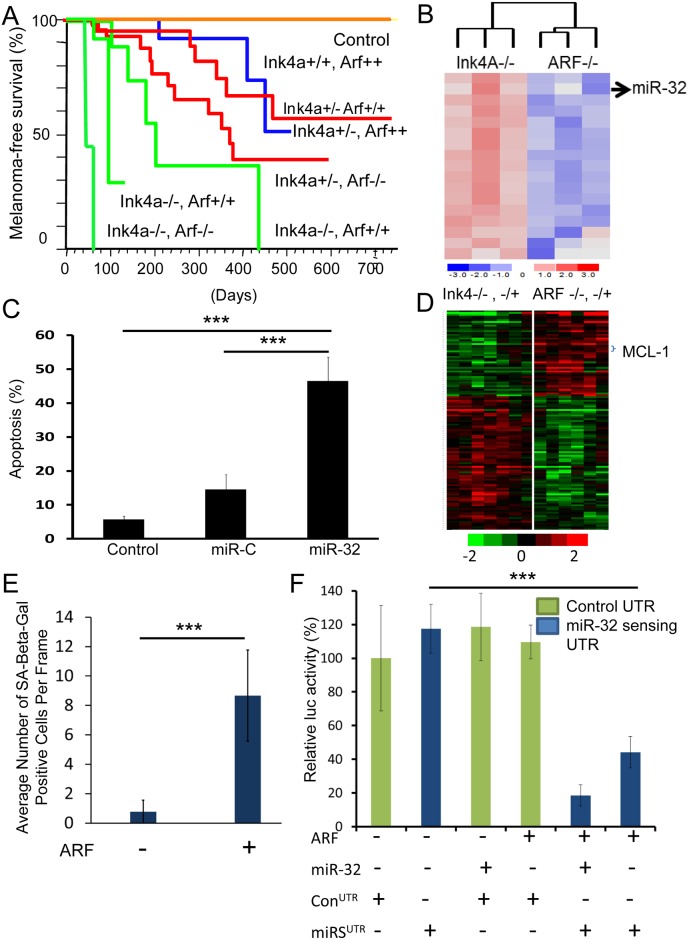
Genomic and microRNAomic analysis identifies *miR-32* and *MCL-1* as critical players in melanomagenesis. A, UV-initiated melanomas driven *in vivo* by a hepatocyte growth factor/scatter factor (HGF/SF) transgene (2, 3) arose with higher frequency and shorter latency when deficient in *ARF* relative to *INK4a*. Non-transgenic mice were used as control. B, miRNA expression array was performed on *NRAS*-transformed *ARF*^-/-^ and *INK4a*^-/-^ melanocytes, which identified *miR-32* as one of the top miRNAs upregulated in *ARF*-/- *NRAS* cells. C, Apoptosis was quantitated using TUNEL assay on *ARF*^-/-^*NRAS* cells alone (control), transfected with control miRNA (miR-C) or *miR-32*. ***P<0.001, as determined by students t-test. D, Gene expression analysis of *ARF*-deficient and *INK4a*-deficient melanomas from the HGF/SF transgenic mice (S1 Fig) identified *MCL-1* as one of the top overexpressed genes associated with *ARF* loss. The complete gene list is presented in S1A Fig; the principal component analysis is shown in S1B Fig; the GO analysis is presented in S1 Table; and a gene network analysis of the top over-represented genes in tumors lacking *ARF* is presented in S2 Fig, Senescence was quantitated by re-expression of ARF in the ARF-/- melanocytes. F. miR-Sens- technology was used to detect miR-32 activity [24] using a miR-sensing UTR (with a miR-32 binding site) in ARF-/- cells. A mutant UTR, where miR-32 site was deleted was used as a control. ***P<0.001, as determined by students t-test.

A key to improving treatment options for melanoma patients lies in a better understanding of mechanisms underlying melanoma progression, which are complex and heterogeneous. In this regard, tractable genetically engineered mice (GEM) that accurately model the genesis and progression of human melanoma can be critical tools for experimental analysis and discovery. The MET-deregulated hepatocyte growth factor/scatter factor (HGF/SF)-transgenic mouse model develops UV-dependent tumors that resemble human melanomas with respect to histopathology, etiology and molecular wiring [7]. We have discovered that UV-initiated melanomas driven by the *HGF/SF-*transgene arise with a significantly higher frequency and shorter latency when deficient in *ARF* relative to *INK4a* (Fig 1A) [8, 9]. Although less well studied compared to *INK4a*, *ARF* is a frequent target of deletion, inactivating mutation or methylation suppression in human melanoma [10–12].

In this study we elected to exploit the differences in malignancy associated with these two tumor suppressors to elucidate mechanisms underlying melanoma progression. We utilized gene and microRNA (miRNA) expression data from INK4a*-* and ARF-associated melanoma models, as well as available human melanoma databases, to uncover an important role for a pathway centered on the tumor suppressor miRNA *miR-32* and the anti-apoptosis oncogene *MCL-1*. Moreover, our data suggest that their inhibition may be an effective anti-melanoma strategy, irrespective of the status of *NRAS*, *BRAF or PTEN*.

## Materials and Methods

### Ethics Statement

All mouse work was performed with the approval of the NCI Animal Care and Use Committee, in accordance with AALAC guidelines and policies established by the NCI.

### Genetically Engineered Mice

Mice carrying an *HGF/SF* transgene whose expression is regulated by a metallothionein gene promoter and flanking regions were made as described [7]. Mice carrying specific inactivating mutations in *ARF* or *INK4a* were generated as described [13]. Unless otherwise indicated, all melanomas arose from mice on a genetic background consisting of ≈90% FVB/N: 10% C57BL/6. All melanomas were initiated by a single dose of UV radiation at 3.5 days of age as described [14].

### miRNA Expression Profiling using miRNA-microarray

miRNA was isolated using miRCURY RNA Isolation kit (Exiqon) as per manufacturer’s protocols. The miRNA profiling was performed using LNA^™^ enhanced microarrays (Exiqon). Briefly, the sample quality control was performed on the Bioanalyser2100 and RNA measurement was performed on the Nanodrop instrument. The samples were labeled using the miRCURY^™^ Hy3/Hy5 Power labeling kit and hybridized on the miRCURY^™^ LNA Array (version 5th Generation arrays, mmu). The samples were hybridized on a hybridization station and scanned. The quantified signals (background corrected) were normalized using the global Lowess (LOcally WEighted Scatterplot Smoothing) regression algorithm, within-slide normalization to minimize the intensity-dependent differences between the dyes. *miR-32* levels were further validated by qRT–PCR using LNA based proprietary primers and probes (Exiqon).

### Gene Expression Profiling using Microarray

Gene expression analysis of the ARF-deficient and INK4a-deficient melanomas arising in our HGF/SF transgenic mice was performed. All melanomas arose from mice on a genetic background consisting of ≈90% FVB/N: 10% C57BL/6. All melanomas were initiated by a single dose of UV radiation to new born mice at 3.5 days of age using previously described methods [15]. Briefly, RNA was isolated using TRIzol reagent, according to the manufacturer's instructions (Invitrogen), and quantified fluorimetrically using an Agilent Bioanalyser (Agilent Technologies).vBiotinylated cRNA were prepared according to the standard Affymetrix protocol. Affymetrix GeneChip Mouse Genome 430 2.0 Array [Mouse430_2] was scanned using Affymetrix standard scan protocol. Data was analyzed using GeneSpringGX using following parameters: Filtered expression (20.0); Mann-Whitney unpaired p-value cut-off > 0.05. The gene expression data has been deposited to GEO database with accession number GSE87331.

### TUNEL Assay to Measure Apoptosis

To quantitate apoptosis a TUNEL assay was performed using TACS TdT Fluorescein kit (R&D Systems) as per the manufacturer’s protocol.

### Transfections of *miR-32* and siRNAs

Melanocytes were plated in six or 12 well plates and 24 h later transfected with 100nM of either *miR-32*, non-specific miRNA or *MCL-1* siRNA using oligofectamine (Invitrogen) [16] according to the manufacturer's protocols. Cell survival rate was very high upon transfections (over 90%) as no significant cell death was observed after cell transfections. Transfection efficiency was significantly high as measured by specific down regulation of miR-32 target genes.

### Melanocyte Derivation, Culture, and Growth Curves

*ARF-/-* and *INK4a-/-* melanocytes were derived from mice with >95% C57BL/6J background. Primary melanocyte cultures were prepared from neonatal skins as described by using a feeder layer of mitomycin-treated immortal murine XB2 keratinocytes for the first two passages only [17]. Cultures were grown at 37°C in complete melanocyte growth medium (CMGM) (RPMI medium 1640 with 10% FBS, 200nM 12-O-tetradecanoyl phorbol 13-acetate, and 200pM cholera toxin) [18]. The cells were grown in a CO2 incubator with 10% CO2 and pH 6.9–7.0. Medium was changed twice a week.

### Proteasome Inhibitor Assay

Proteasome inhibitor assay was performed as described previously [18]. *ARF* was re-expressed in *INK4a*-*ARF*^*-/-*^ melanocytes by infection with a retrovirus containing *ARF* cDNA. 95% ethanol was used to dissolve LLnL (Sigma–Aldrich). Melanocytes were treated with a final concentration of 50μM LLnL in media for 16h. Melanocytes were rinsed with PBS and harvested for analysis after individual treatment.

### Vectors and Generation of Modified Cell Lines

The 3' UTRs of *MCL-1* (*MCL-1*wt and *MCL-1*mut), *PIK3R3*wt and *NRAS* (*NRAS*wt and *NRASmut*) were cloned in to pEZX-MT01 vector backbone and the mutations were generated by site directed mutagenesis (GeneCopoeia Inc.). WM3928 cell line was chosen for this experiment because it is wild-type for BRAF and wild-type for NRAS genes and unlike other mutant RAF-RAS expressing cells, RAS/RAF pathway is least active. These wt and mutant vectors were transfected into the WM3928 melanoma cell line, which was in turn transfected with *miR-32* mimics. The expression of the reporter gene, driven by a wt or mutant 3’UTR, was determined using luciferase assay. Briefly, cells were plated in 12-well plates. After 24 hours cells were transfected with *miR-32* mimics (100nM) using oligofectamine (Invitrogen) according to the manufacturer's protocols. Cell lysates were made, and luciferase assays were carried out using the luciferase assay system kit (Promega) as per the manufacturer's protocol. Briefly, cells were lysed using 80μL lysis buffer, the cell lysates were centrifuged at 14,000 rpm, and the supernatant was collected. Twenty-microliter supernatant was mixed with 100μL of luciferin, and luciferase activity was measured using a luminometer (GloMax 20/20, Promega). Three individual repeats of each experimental condition were performed.

### Immunoblot Analysis

Immunoblot analysis was performed by using SDS/PAGE followed by Western blot analysis. Equal loading was determined by using Ponceau S and anti-α-tubulin antibody. Primary antibodies used were *MCL-1* (D35A5, mAb #5453, Cell Signaling Technology), *p19ARF* (ab80, Novus Biologicals Inc. Littleton, CO) and α-tubulin (mAB # B-5-1-2, Sigma Chemical Co). The data were analyzed using the Image-J software from the National Institutes of Health (Bethesda, MD).

### Soft Agar Assay

Anchorage-independent growth was assayed using previously described methods [19] by plating 5×10^4^ cells per well mixed with soft agar in 6-well plates. After 4–6 weeks incubation the cells were fixed with methanol and acidic acid solution (10%), and stained with ethanol (20%), crystal violet (0.4%); large and small colonies were scored. All experiments were done in triplicate.

### Sabutoclax and Vemurafenib Cytotoxicity

Sabutoclax and vemurafenib cytotoxicity was carried out in RPMI medium 1640 with 10%, penicillin/streptomycin, and L-glutamine. A375P cell line was chosen for this study because it is mutant for BRAF (NRAS wild-type/PTEN wild-type) to mimic mutant BRAF melanomas. Out of three available A375 lines (low-A375P, medium-A375M, and high-A375SM metastatic and invasive properties), low-A375P line was chosen due to its low metastatic and invasive properties. The A375P melanoma cells were cultured in the RPMI media for several passages before plating for the cytotoxicity assay. The [3-(4,5-dimethylthiazol-2-yl)-5-(3-carboxymethoxyphenyl)-2-(4-sulfophenyl)-2H-tetrazolium, inner salt] (MTS) (Promega, Madison, WI) was used to assay the cytotoxicity according to the Cell-Titer 96 aqueous one-solution protocol. Two thousand cells were plated in each well of 96-well plates in 180μl of the medium. After 24h, varying concentrations of sabutoclax and vemurafenib and their combinations in 20μl of media were added and cells were incubated for 96h. After 96h of incubation, the MTS assay was performed and the absorbance in each well was determined at 490nm by using a plate reader. Wells containing cells with no drug and wells with medium alone were used as positive and negative controls, respectively. All experimental points were set up in replicate wells, and all experiments were repeated four times. Combination index values were determined using Chou-Talalay method for drug combination using CalcuSyn version 2 (Biosoft).

### In Vivo Tumor Growth Analysis

In vivo tumor-growth analysis was carried out by implantation into 4-6-week-old immune compromised mice (NCI nu nudes). A375P melanoma cells were transfected with *miR-32* and *siMCL-1* and subcutaneously implanted at 0.5 X 10^6^ cells per injection site per mouse (experimental n = 5, control n = 10). Tumor volume was measured at day 32 after implantation.

### In Vivo Drug Combination and miRNA Combination Study

A375 melanoma xenograft model was used because it is mutant for BRAF (NRAS wild-type/PTEN wild-type) to mimic mutant BRAF melanomas to assess the effects of high-dose vemurafenib (500nM) or sabutoclax (500nM) alone, and low-dose (25nM to 5nM) sabutoclax (S) and vemurafenib (V) combination (n = 5, control n = 10, 0.5 X 10^6^ cells per mice); S+V-1 (V25nM+S25nM), S+V-2 (V12.5nM+S12.5nM) and S+V3 (V5nM+S5nM). The doses were selected based on *in vitro* experiments, ranging between 1-5fold relative sabutoclax and vemurafenib IC-50 values, alone and in combination. Tumor growth was monitored at 4-day intervals. The Kaplan-Meier analysis and log-rank test were used to determine the association of drug treatment to survival. The survival study was performed using humane endpoints using protocols approved by the NCI Animal Care and Use Committee, in accordance with AALAC guidelines and policies established by the NCI. The animals were euthanized when they met a specific criterion. Endpoints included, but are not limited to: a) tumor burden greater than 10% body weight i.e. for an adult mouse, a tumor should not exceed 20 mm in any one dimension; b) lack of responsiveness to stimulation; hunched posture; b) immobility; and/or c) an inability to eat or drink. Standard approved protocols for euthanasia were followed using Carbon Dioxide inhalation methods. There were no unexpected deaths in this study and health of the animals was monitored daily during the lifetime of this study by personnel experienced in recognizing signs of morbidity (illness, injury, or abnormal behavior). If animals appeared lethargic, sick or distressed they were euthanized the same day. Any visible necrosis was taken care by standard medications and all efforts were made to minimize suffering.

Furthermore, the A375P melanoma xenograft model was also used to test whether *miR-32* pretreatment can potentiate the anti-tumor effects of sabutoclax (S) and vemurafenib (V) in combination (n = 5, control n = 10, 0.5 X 10^6^ cells per mice). Dosage used were as follows: si:si*MCL-1* (100nM), miR: *miR-32* (100nM), V: vemurafenib (500nM), S: sabutoclax (500nM); combination treatment with miR+V: *miR-32* (100nM) and vemurafenib (25nM); miR+S: *miR-32* (100nM) and sabutoclax (25nM); S+V: sabutoclax (25nM) and vemurafenib (25nM); miR+S+V: *miR-32* (100nM), sabutoclax (25nM) and vemurafenib (25nM). Tumor volume was measured at day 32 after treatment.

### Statistical Analysis

All statistical analyses were performed using Graphpad Prism software. All experiments were repeated at least twice. Statistical significance and p-values were determined by one-way analysis of variance (ANOVA) or student's t test. Means ± s.e.m. are indicated for all statistical analyses. A p-value of <0.05 was considered statistically significant.

## Results

### The microRNA/mRNA pair *miR-32*/*MCL-1* is deregulated in malignant melanoma

**Fig 2:**
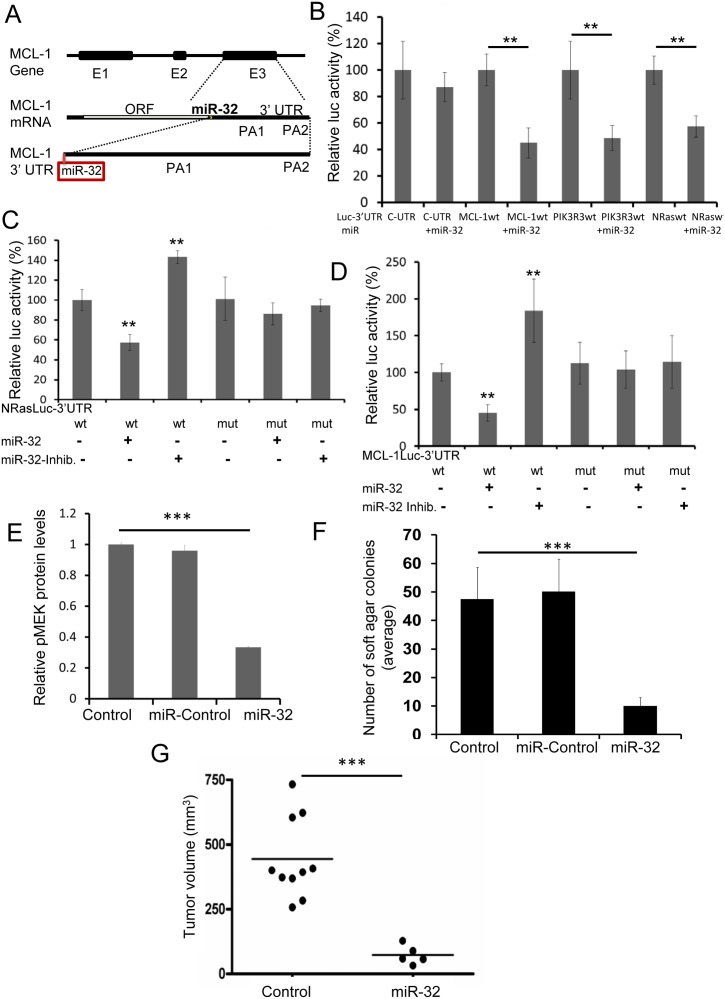
*miR-32* inhibits melanomagenesis in part by regulating the expression of the *NRAS*, *PI3K* and *MCL-1*. A, *MCL-1* 3’UTR harbors a well-conserved 8-mer binding site for *miR-32* in exon 3. The *miR-32* targets were predicted using a GOmiR algorithm [22]. E1-3 represents exons 1–3; polyadenylation sites are abbreviated as PA. The complete analysis as well as prioritization of all the miRNA binding sites in *MCL-1* 3’UTR are presented in S2 Table. B, The 3’UTRs of *NRAS*, *PIK3R3*, *MCL-1* and control-UTR (C-UTR, not regulated by miR-32) regulating luciferase reporter gene expression were transfected into the WM3928 melanoma cell line followed by transfection with *miR-32* mimics. The expression of the reporter genes was determined through luciferase assays. **P<0.01, as determined by students t-test. C, A *miR-32* seed region deletion mutant was created using site-directed mutagenesis. The wildtype-*NRAS* 3′UTR (*NRAS*wt) and mutant-*NRAS* 3’UTR (*NRAS*mut) were transfected into WM3928 cells followed by transfection with *miR-32* mimic and inhibitor. **P<0.01, as determined by students t-test. D, Wildtype-*MCL-1* 3′ UTR (*MCL-1*wt) and mutant-*MCL-1* 3’UTR (a *miR-32* seed region deletion mutant in *MCL-1* 3’UTR, *MCL-1*mut) were transfected into WM3928 cells followed by *miR-32* mimic and inhibitor transfection. **P<0.01, as determined by students t-test. We also found that the *miR-32* gene is highly conserved between species and frequently downregulated in human cancers and hence, can be a strong candidate tumor suppressor miRNA (see Fig 3 and S3 Fig). E, MAPK pathway activation was quantified using pMEK protein levels by western blotting and imageJ respectively. ***P<0.001, as determined by one-way analysis of variance (ANOVA) indicating that miR-32 over expressing cells express low pMEK levels. F. Anchorage-independent growth was measured by soft agar colony forming assay. **P<0.01, as determined by students t-test. G, A375 control and *miR-32* overexpressing cells were transplanted subcutaneously into immune compromised mice; tumor growth was measure at day 32. **P<0.01, as determined by students t-test.

We began our analysis using a simplified model in which we examined the behavior of *INK4a*-deficient and *ARF*-deficient mouse melanocytes infected with a retrovirus encoding oncogenic *NRAS* (*NRAS*^*Q61K*^), which is mutated in 20% of human melanomas [20, 21]. As in autochthonous GEM melanomas, transformed *ARF*-deficient melanocytes exhibited a much more aggressive phenotype relative to those deficient in *INK4a*^*-/-*^ [18, 21]. Comprehensive transcriptomic expression analysis was performed on *NRAS*-transformed *INK4a*^*-/-*^ and *ARF*^*-/-*^ melanocytes. A miRNA signature specifically associated with melanomagenesis mediated by *ARF loss* was identified, with *miR-32* as one of the top miRNAs upregulated in *NRAS*-transformed *ARF*^*-/-*^ cells; miRNA had not previously been implicated in melanoma (Fig 1B). Overexpression of *miR-32* in *NRAS*-transformed ARF^*-/-*^ malignant melanocytes induced apoptosis, suggesting a role for *miR-32* in regulating cell survival (Fig 1C). A GO analysis of *miR-32*-predicted target genes identified apoptosis as one of the biological processes regulated by *miR-32*. Using a GOmiR algorithm [22] to integrate data from various target prediction algorithms, such as miRanda, TargetScan, PicTar4way, RNAhybrid, TraBase and PicTar5way, we discovered that *miR-32* has a binding site in the 3’ UTRs of induced myeloid leukemia cell differentiation protein (*MCL-1*), an early critical negative regulator of apoptosis [23] (Fig 2A and S2 Table), as well as its upstream effectors *NRAS* and *PIK3R3* (the regulatory subunit of *PI3K*). Notably, gene expression analysis of the *ARF*-deficient and *INK4a*-deficient melanomas arising in our *HGF/SF* transgenic mice also identified *MCL-1* as one of the top overexpressed genes associated with more malignant melanomas (Fig 1D and S1A Fig). Principal component analysis revealed that the *ARF*-deficient samples clustered together, as did the *ARF*-deficient melanomas (S1B Fig). A GO analysis revealed that loss of *ARF*, unlike *INK4a*, is associated with upregulation of GO groups linked to proliferation, DNA synthesis and repair machinery (S1A and S1B Table). A gene network analysis of the top over-represented genes in tumors lacking *ARF* linked *MCL-1* to NFκB, interferon alpha, RNA polymerase II, follicle-stimulating hormone and cyclin D2 pathways (S2 Fig). Re-expression of ARF induced senescence (Fig 1E) in the ARF-/- melanocytes. A luciferase vector-based approach (miR-Sens-technology) was used to accurately detect miR-32 activity in these cells [24]. We found that miR-32 activity was significantly induced upon ARF re-expression, as evidenced by the fact that down regulation of the miR-sensing UTR was comparable to miR-32 transfected cells (Fig 1F).

### *miR-32* regulates the expression of *MCL-1*, *PIK3R3*, *NRAS* and signaling through the MAPK pathway

To validate the predicted miR-targets, the 3’UTRs of *NRAS*, *PIK3R3* and *MCL-1*, regulating luciferase reporter gene expression, were transfected into the WM3928 melanoma cell line (wildtype for both *NRAS* and *BRAF*). *miR-32* mimics were transfected into the WM3928 cells expressing the *NRAS*, *PIK3R3*, or *MCL-1* 3’UTRs. The expression of the reporter genes was determined through luciferase assays. Overexpression of the *miR-32* mimic led to down-regulation of luciferase protein driven by the *NRAS*, *PIK3R3*, and *MCL-1* 3’UTRs (Fig 2B).

To confirm that the *miR-32* target site in the 3’UTR of *NRAS* was a true site, we created a deletion mutant that completely abolished the seed region of the *miR-32* binding site in the *NRAS* 3’UTR. The wildtype-*NRAS* 3′UTR and mutant-*NRAS* 3’UTR were transfected into WM3928 cells. *miR-32* mimics were then transfected into wildtype-*BRAF* and mutant-*NRAS* 3′UTR-expressing WM3928 cells. Overexpression of the *miR-32* mimics in cells containing the wildtype *NRAS* UTR led to down-regulation of reporter protein (Fig 2C). In contrast, the *miR-32* mimic had no effect on the expression level of reporter protein in cells overexpressing the mutant UTR, indicating that *miR-32*-mediated regulation of *NRAS* was lost in mutant-*NRAS* 3’UTR-expressing cells. Hence, *miR-32*-mediated regulation requires an intact *miR-32* binding site in *NRAS* 3′UTR (Fig 2C), suggesting that *NRAS* expression is regulated by *miR-32*.

We went on to determine experimentally if the *miR-32* target site in the 3’UTR of *MCL-1* was a true site. The *MCL-1* 3’UTR harbors a well-conserved 8-mer binding site for *miR-32*. *MCL-1* mRNA harbors two polyadenylation sites (PA1 and PA2) and the *miR-32* binding site is located at the beginning of the *MCL-1* 3’UTR; therefore, *miR-32* would be predicted to regulate all forms of *MCL-1* mRNAs irrespective of polyadenylation site (Fig 2A and S2 Table). A deletion mutant was created that completely abolished the seed region of the *miR-32* binding site in the *MCL-1* 3’UTR. Then the wildtype-*MCL-1* 3′ UTR and mutant-*MCL-1* 3’UTR were transfected into WM3928 human melanoma cells. Overexpression of the *miR-32* mimics in wildtype cells led to down-regulation of *MCL-1*-associated activity (Fig 2D). In contrast, cells overexpressing the mutant *miR-32* mimic had no effect on the expression level of reporter protein, indicating that *miR-32*-mediated regulation of *MCL-1* was lost due to the mutation (Fig 2D). Hence, we conclude that *MCL-1* expression is regulated by *miR-32*.

### *miR-32* is a strong candidate tumor suppressor miRNA

**Fig 3:**
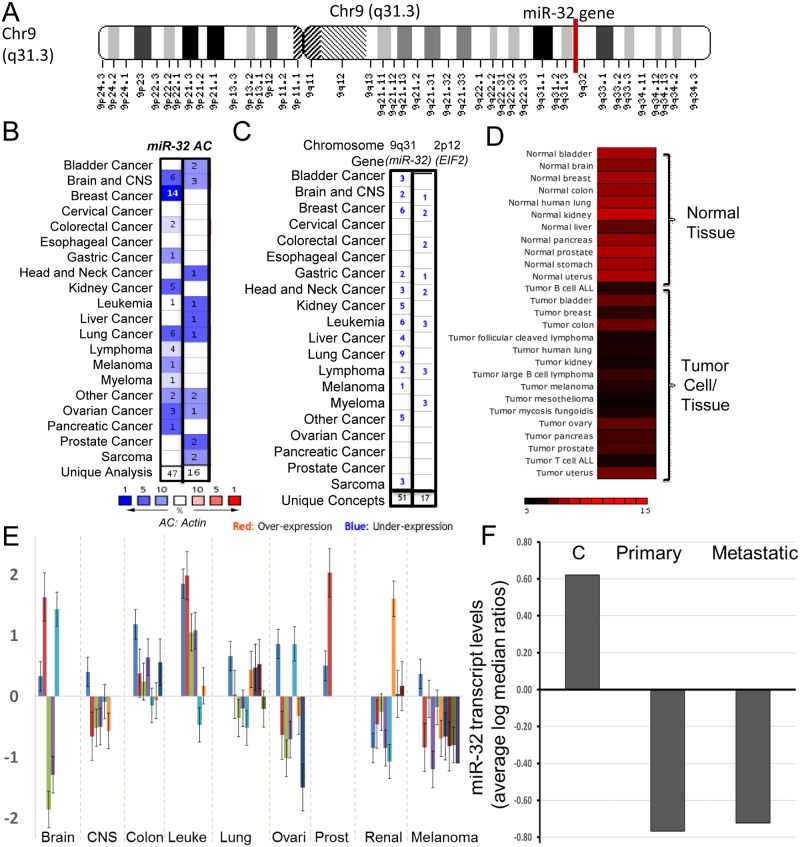
The miR-32 is frequently downregulated in human cancers and is a strong candidate tumor suppressor miRNA. A, miR-32 is located on chromosome 9q31 (in the NR_029506.1 noncoding region) and is highly conserved between species (according to miRcode the miR-32 gene is conserved 89% among primates and 61% among mammals) B, A meta-analysis, using Oncomine algorithm, was performed to measure levels of miR-32 across cancer data sets as compared to normal tissue. Actin expression was used as control for this analysis. C, A chromosomal cytoband meta-analysis was performed on miR-32-cytoband-9q31using normal vs. cancer oncomine datasets. Chromosomal cytoband 2p12 was used as a control for this analysis. D, miR-32 transcriptome levels were assessed using miRNAMap-2 in normal human tissue as compared to various human cancer types. E, miR-32 transcript expression is down regulated in several cancer types including melanoma in NCI 60 cancer cell line panel. F, miR-32 transcript levels are expressed very low levels in primary and metastatic melanoma tumors as compared to melanocytes (C:control).

*miR-32* is located on chromosome 9q31 (Fig 3A and S3 Fig) and is highly conserved between species (according to miRcode the *miR-32* gene is conserved 89% among primates and 61% among mammals). A meta-analysis revealed that *miR-32* was downregulated in 47 cancer data sets as compared to normal tissue. Actin, used as control gene for this analysis, was downregulated in only 16 cancer types (Fig 3B). Furthermore, a chromosomal cytoband meta-analysis showed that cytoband 9q31 was downregulated in 34 cancers (normal vs. cancer analysis). Chromosomal cytoband 2p12, used as a control for this analysis, was downregulated in only 10 cancer types (Fig 3C). *miR-32* was upregulated in normal tissue and downregulated in various human cancer types (Fig 3D). A miR-32 transcriptomics analysis in NCI 60 cancer cell line panel using NCIs CellMiner database [25] revealed that miR-32 expression is down regulated in several cancer types including melanoma (Fig 3E). Moreover, an analysis of miR-32 transcript levels in primary and metastatic melanoma tumors samples revealed that miR-32 is down regulated in primary and metastatic melanoma as compared to melanocytes (Fig 3F).

Hence the *miR-32* gene, highly conserved between species and frequently downregulated in human cancers, including melanoma and is a strong candidate tumor suppressor miRNA. Accordingly, ectopic *miR-32* overexpression in melanoma cells down regulated pMEK levels (Fig 2E) induced apoptosis (Fig 1C), and reduced anchorage-independent growth (Fig 2F) and tumor growth *in vivo* (Fig 2G), suggesting that *miR-32* acts as a tumor suppressor by inducing apoptosis and reducing the tumorigenicity of melanoma cells.

### *MCL-1* is a strong candidate melanoma oncogene

**Fig 4:**
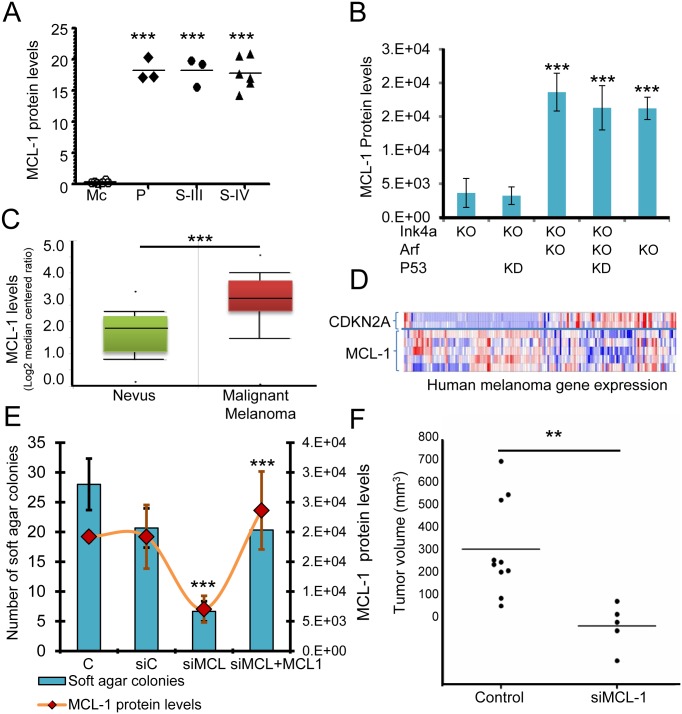
*MCL-1* acts as a critical player in melanomagenesis. A, *MCL-1* protein levels were measured and quantified in human melanocytes (Mc), primary tumor (P), and patient-derived human metastatic melanoma cell lines—stage III (S-III) stage IV (S-IV) subtypes using western blotting and imageJ respectively. ***P<0.001, as determined by one-way analysis of variance (ANOVA). B, *MCL-1* levels were measured and quantified in mouse melanocytes that were *INK4a* -/- (KO), *ARF*-/- (KO), and in which p53 was knock down (KD) using a shRNA (2). ***P<0.001 was determined by ANOVA). C, *MCL-1* mRNA levels were upregulated in human malignant melanomas as compared to nevi. A meta-analysis, demonstrating that *MCL-1* is highly upregulated in 57 cancer data sets as compared to normal tissue, is presented in S4D Fig, CDKN2A and *MCL-1* transcript expression were quantified in human melanoma clinical samples. E, *ARF*^-/-^ *NRAS* transformed mouse cells were transfected with a control siRNA (siControl) and a siRNA that targets *MCL-1* (siMCL1). Rescuing MCL-1 expression by overexpressing MCL-1 in siMCL1 transfected cells restored colony-forming ability of the siMCL1 cells suggesting that the anchorage independent phenotype can be attributed to MCL-1 expression. *MCL-1* protein levels (orange line) and soft agar colony forming ability was quantified. ***P<0.001, as determined by students t-test. F, *ARF*^-/-^ *NRAS* control and *ARF*^-/-^ *NRAS* si*MCL-1* transfected cells were transplanted subcutaneously in immunocompromised mice (NCI nu nudes); tumor growth was measure at day 32. **P<0.01, as determined by students t-test.

To begin to understand the role of *MCL-1* in human melanomagenesis, a meta-analysis was used to show that compared to normal tissue *MCL-1* was highly upregulated in 57 cancer data sets, whereas *E2F-1*, as a positive control, was upregulated in 45 cancer data sets (S4 Fig). Actin was used as a negative control for this analysis and was upregulated in only 8 cancer types. *MCL-1* protein was found to be expressed at low levels in human primary melanocytes; however, it was expressed at high levels (over 20-fold) in patient-derived melanoma cell lines (primary tumor and stage II/III metastatic melanoma) (Fig 4A). *MCL-1* was upregulated in human malignant melanoma as compared to nevi (Fig 4C). In mice, *MCL-1* levels were elevated in *NRAS*-transformed *ARF*^-/-^ cells (Fig 4B). We observed an inverse correlation between expression of *CDKN2A* gene transcripts and *MCL-1* transcripts in human melanoma clinical samples (*INK4a* and *ARF* are not separable in this analysis) (Fig 4D). To confirm that high levels of *MCL-1* can drive cellular transformation, we knocked down *MCL-1* in *NRAS*-transformed *ARF*^-/-^ cells using a siRNA (*siMCL1*) that targets *MCL-1* mRNA and protein levels. The siRNA markedly reduced the levels of *MCL-1* protein in these cells, resulting in a significant reduction in soft agar colony forming ability (P<0.05) (Fig 4E) and tumor growth *in vivo* (Fig 4F). Rescuing MCL-1 expression by overexpressing MCL-1 in siMCL1 transfected cells restored colony forming ability of the siMCL1 cells, indicating that the anchorage independent phenotype can be attributed to MCL-1 expression (Fig 4E). These data suggest that increased *MCL-1* levels are indeed required for the transformed phenotype.

### *ARF* can induce proteasomal-mediated degradation of *MCL-1* and induce apoptosis

**Fig 5:**
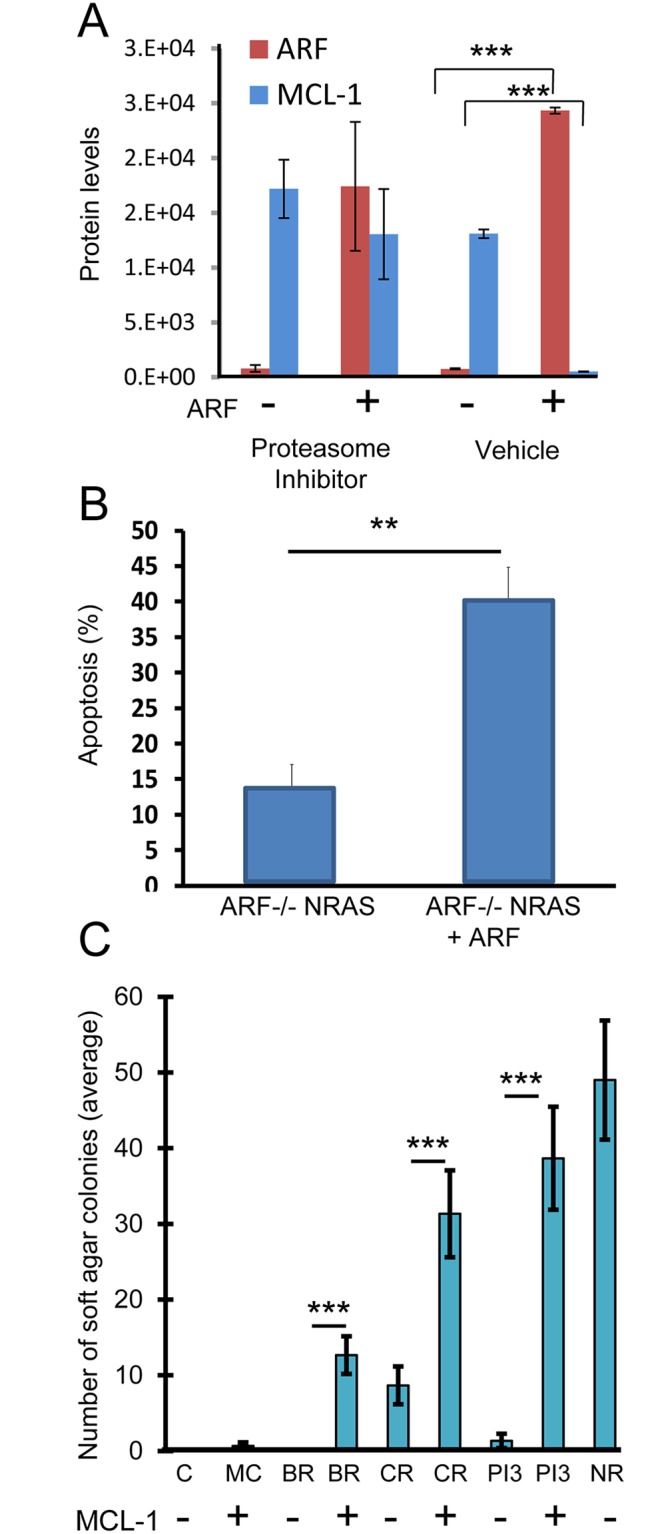
*MCL-1* can be degraded by *ARF* re-expression and can cooperate with *BRAF*^V600E^, CRAF, and PI3K to induce cellular transformation. A, A proteasomal degradation assay was performed using *ARF*^-/-^ *NRAS* cells transfected with an *ARF*-containing vector in the presence and absence of the proteasomal inhibitor, LLnL. ***P<0.001, as determined by students t-test. B, Apoptosis was quantitated in *ARF ARF*^-/-^ *NRAS* mouse cells expressing *ARF*. **P<0.01, as determined by students t-test. C, *MCL-1* was expressed in *ARF*-deficient mouse melanocytes expressing mutationally activated forms of *BRAF* (V600E) (BR), CRAF (CR) or PI3K (PI3) [21]. Soft agar colony forming ability of control (C) and MCL-1 overexpressing (MC) cells was determined. *ARF*-/- *NRAS* (NR) cells were used as positive control. ***P<0.001, as determined by students t-test.

Since *MCL-1* levels were greatly enhanced in *ARF*-deficient melanocytes as well in metastatic melanomas relative to wildtype (Fig 4B–4D), we investigated the possibility that *ARF* can regulate *MCL-1* expression. Since *ARF* is known to induce proteasomal protein degradation [18, 26], a proteasomal degradation assay was performed to determine if *MCL-1* is a target of *ARF*-mediated proteasomal degradation. *ARF* was re-expressed in *ARF*-deficient melanocytes in the presence and absence of a proteasomal inhibitor. *ARF* re-expression decreased the level of *MCL-1*; however, this effect was abrogated in the presence of the proteasomal inhibitor, LLnL (Fig 5A). Additionally, re-expression of *ARF* reduced cell growth and induced apoptosis (Fig 5B). These data raise the possibility that *ARF* down-regulates *MCL-1* levels through a proteasomal-mediated degradation mechanism that could account, at least in part, for its ability to trigger apoptosis in melanocytes.

### *MCL-1* can co-operate with *BRAF* and PI3K activation in oncogenic transformation

Next, we explored the effects of forced *MCL-1* upregulation in *ARF*-deficient melanocytes. We were able to achieve ~8-fold overexpression of MCL-1 using the vector in ARF-/- primary melanocytes. We found that forced *MCL-1* overexpression alone was unable to transform *ARF*-deficient cells, which grew in a monolayer, morphologically resembled the parental cells, and failed to grow in soft agar. However, when forced *MCL-1* overexpression in *ARF*-deficient melanocytes was combined with expression of mutationally activated forms of *BRAF* (V600E), CRAF or PI3K [21], numerous transformed phenotypes became apparent, including morphologic changes (refractile, spindle shaped), loss of contact inhibition, and enhanced growth in soft agar (Fig 5C). These results indicate that *MCL-1* can co-operate with *MAPK* and *PI3K* activation in oncogenic transformation, and raise the possibility that *MCL-1* may be an effective secondary target when designing combination therapies.

### *miR-32* replacement therapy and the *MCL-1*-specific antagonist sabutoclax were effective as single agents, and acted synergistically in combination with vemurafenib

Melanocytes are intrinsically resistant to apoptosis, which is thought to help explain the characteristically chemo-resistant behavior of melanoma [27]. To test the hypothesis that expression of *MCL-1*, a known anti-apoptotic factor, can contribute to drug resistance, we targeted *MCL-1* using a small molecule inhibitor (sabutoclax) in a variety of human melanomas, including *BRAF*^V600E^ and non-*BRAF* stage III/stage IV metastatic melanoma cell lines (Table 1). Sabutoclax is designed to specifically inhibit the BH3 binding domain of MCL-1 [28, 29] and has an MCL-1-selective activity in prostate cancer cells [30]. We found that sabutoclax treatment was very effective not only in *BRAF*^V600E^-expressing lines, regardless of *PTEN* status, but also in non-*BRAF* melanomas that currently have no effective clinical options (Table 1).

**Table 1:** A *MCL-1* specific inhibitor sabutoclax treatment is very effective not only in *BRAF*^V600E^-expressing lines, but also in non-*BRAF* melanomas that currently have no effective clinical options in the clinic.

Melanoma	Gender/age	Stage/site	*BRAF*/*NRAS* mutation	*PTEN*	Vemurafenib		Sabutoclax	
					Average IC-50 (μM)	STDEV	Average IC-50 (μM)	STDEV
Wild-type for *BRAF* and *NRAS*								
YUKIM	F/71	III, lymph node	WT	WT, LOH	6.615	0.422	0.523	0.026
WM3928		Metastatic melanoma	WT	unknown	6.329	0.337	0.346	0.008
*BRAF* mutants/*NRAS* wild-type/*PTEN* wild-type								
WM88	unknown	IV, metastatic	V600E	WT	0.070	0.012	0.238	0.007
A375	F/54	IV, lymph node metastasis	V600E	WT	0.131	0.006	0.186	0.003
*BRAF* mutants/*NRAS* wild-type/ *PTEN* mutant								
YUGEN8	F/44	IV, brain	V600E	Null	0.134	0.017	0.105	0.002
YULAC	F/66	IV, soft tissue, neck	V600K	P38S/LOH (C1143T)	0.127	0.019	0.070	0.011
*NRAS* mutants/*BRAF* wild-type								
YUDOSO	M/84	llb, primary melanoma, left abdomen, 2.5 mm	Q61K/WT	WT	6.839	0.238	0.346	0.008
WM3066		IV, metastatic	Q61K	WT	7.934	2.432	1.038	0.063

F: female; M: Male; II: Stage II, III: stage III and IV: stage IV metastatic melanoma; WT: wild type; Null: lacking the gene of interest; LOH: loss of heterozygosity.

**Fig 6:**
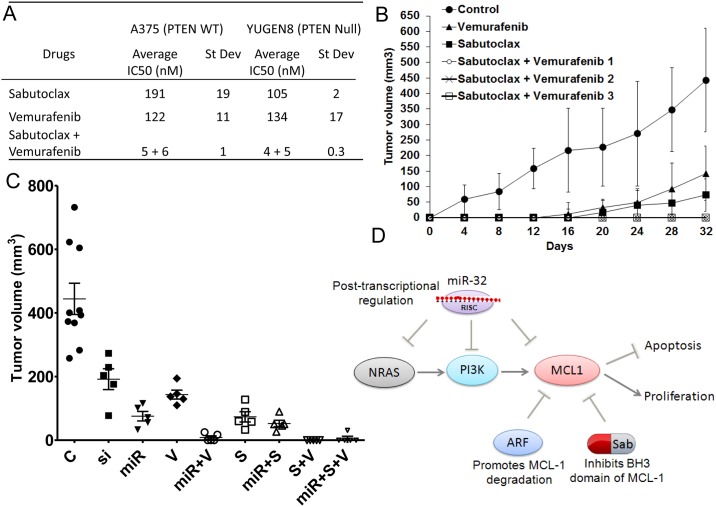
The *MCL-1*-specific antagonist sabutoclax and *miR-32* were effective as single agents, and acted synergistically in combination with vemurafenib in preclinical melanoma models. A, Synergistic effects of the *MCL-1* inhibitor sabutoclax with vemurafenib were tested by the Chou-Talalay method using the melanoma cell lines A375 (*BRAF*^V600E^/*PTEN* WT) and YUGEN8 (*BRAF*^V600E^/*PTEN* Null). Average IC-50 values from four different experiments are presented along with standard deviations. Combination index analysis is presented in S3 Table. B, Addition of low-dose (25nM to 5nM) sabutoclax and vemurafenib in combination and high-dose sabutoclax alone (500nM) and vemurafenib alone (500nM) resulted in a significant reduction in tumor size in the A375 melanoma xenograft model (n = 5, control n = 10); S+P-1 (V25nM+S25nM), S+P-2 (V12.5nM+S12.5nM), S+P3 (V5nM+S5nM). The association of drug treatment to survival was determined using Kaplan-Meir analysis and log-rank test, presented in S5 Fig ***P<0.001, as determined by students t-test. C, *miR-32* (miR) pretreatment potentiated the anti-tumor effects of sabutoclax (S) and vemurafenib (V) in combination in the A375 melanoma xenograft model (n = 5, control n = 10). ***P<0.001 was determined by ANOVA. Si: treatment with *MCL-1* siRNA. D, A model of the *miR-32*/*MCL-1* pathway in melanoma and perhaps other tumors is presented. The oncogenic *MCL-1* and its upstream effectors *NRAS/PI3K* are regulated by the tumor suppressor activity of both *miR-32*. MCL-1 levels are also regulated by *ARF* mediated proteasomal degradation. *MCL-1* inhibition by *miR-32* or by sabutoclax (Sab), alone or in combination, can be an effective anti-melanoma therapy.

To improve the efficacy and reduce the toxicity of the standard of care [31] we tested if the *MCL-1* inhibitor would be effective in treating *BRAF*^V600E^ tumors in combination with vemurafenib at MTD and sub-MTD doses. We found that combining sabutoclax with vemurafenib resulted in an enhanced effect, even when the *BRAF* inhibitor was used at a lower dose (Fig 6A). The two were synergistic in activity, and together lowered the IC-50 values dramatically, regardless of *PTEN* status (Fig 6A and S3 Table). Next we tested if *MCL-1* inhibition can potentiate the anti-tumor effects of vemurafenib *in vivo*. Addition of low-dose sabutoclax (25nM to 5nM) and vemurafeninb (25nM to 5nM) resulted in a significant reduction in tumor size in the A375 melanoma xenograft model (Fig 6B). Notably, the combination of sabutoclax and vemurafenib worked synergistically, and as well as high-dose vemurafenib (500nM) or sabutoclax (500nM) alone, resulting in a significant delay in melanoma tumor growth, even with low doses of each (25nM to 5nM).

The Kaplan-Meier analysis and log-rank test were used to determine the association of drug treatment to survival. Humane endpoints were used for the animal survival study (see methods for details). No significant difference was observed between the control and vemurafeninb-treated groups in terms of overall survival, with a 50% survival of 60 days. Mice treated with sabutoclax lived somewhat longer with a 50% survival of 110 days (S5 Fig). In contrast, mice treated with sabutoclax plus vemurafeninb lived longer than one year (S5 Fig, p-value < 0.0007). We also discovered that *miR-32* pretreatment can potentiate the anti-tumor effects of sabutoclax and vemurafenib *in vivo* (Fig 6C). Treatment with *miR-32* in combination with sabutoclax and vemurafenib caused a significant reduction in tumor size in the A375 melanoma xenograft model, resulting in a significant delay in melanoma tumor growth. No other toxic side effects besides a transient 5–10% weight loss were noted in the group of animals treated with sabutoclax either alone or in combination. Presumably, more dramatic effects on tumor cell growth would be obtained with additional doses of *miR-32* and these drugs.

## Discussion

A major challenge in developing effective anti-melanoma therapy has been to identify approaches that target multiple melanoma subtypes irrespective of the status of powerful oncogenic drivers, such as mutant *BRAF*, mutant *NRAS*, or loss of *PTEN*. Melanomas tend to be inherently resistant to conventional therapies, resulting in a high incidence of mortality as melanoma accounts for 75% of all deaths associated with skin cancer. Moreover, there are no effective treatment options available for wildtype *BRAF*/*NRAS* melanomas, which constitute ~30% of all melanomas.

During our analysis of the *CDKN2a* locus in malignant melanoma we discovered that upregulation of the pro-survival gene *MCL-1* was associated with loss of *ARF*, a relatively common occurrence in melanoma [12], providing some explanation for the characteristically chemo-resistant behavior of most melanomas. This finding prompted an in depth analysis of the mechanisms by which *MCL-1* is regulated. We report here for the first time that *miR-32* behaves as a tumor suppressor miRNA in melanoma by inhibiting the expression of *MCL-1*, as well as its upstream effectors *NRAS* and PI3K. In this way *miR-32* also helps regulate the *RAS→RAF→MEK→ERF* and *PI3K→AKT→mTOR* pathways. miR-32 is recently shown to target EZH2 and is down regulated in uveal melanoma [32]. Although *MCL-1* overexpression alone was insufficient to transform primary melanocytes, we found that the overexpression of *MCL-1* even at levels 8-fold higher than melanocytes (compared to the ~20-fold higher levels observed in melanoma cell lines) can transform *BRAF*^V600E^-expressing primary melanocytes, and collaborate with *CRAF* and *PI3K* activation to induce anchorage-independent growth in primary melanocytes.

Given the crosstalk demonstrated by our data and the findings reported in the literature, we reasoned that co-targeting *BRAF*^V600E^ and the *MCL-1* pathway might represent an effective combination anti-melanoma strategy. In support of this notion *BRAF*^V600^ signaling has been reported to trigger apoptotic resistance in melanoma by selectively affecting the expression of the *MCL-1* splice variant *MCL-1L* [33]. Moreover, *MCL-1* expression is upregulated upon activation of the *PI3K→AKT* pathway [34], which has been implicated in resistance to *BRAF* inhibitors [35], and downregulated upon inhibition of the MAPK pathway [36]. *BRAF*^V600^ has been shown to not only stabilize the *MCL-1* protein [37], but to induce *MCL-1* transcription through activation of STAT3, rendering melanoma cells susceptible to apoptosis via STAT3 suppression [37]. Finally, depletion of *MCL-1* promotes anoikis in *BRAF*^V600^ melanoma cells [38].

We found that unlike vemurafenib, which is only effective in patients harboring *BRAF*^V600E^ mutant melanomas, targeting *MCL-1* using the small molecule inhibitor sabutoclax is effective in a variety of human melanoma molecular subtypes, including *BRAF*^V600E^ and non-*BRAF* stage III/stage IV metastatic melanoma cell lines (Table 1). Single agent activity of sabutoclax was slightly more effective in *PTEN* null cell lines (105nM) as compared to *PTEN* wildtype cell lines (191nM), whereas vemurafenib treatment showed no differential effects with respect to *PTEN* status. Combining sabutoclax with vemurafenib proved to be highly effective and highly synergistic (summarized in Fig 6D) Due to this strong synergy, efficacy can be achieved by combining vemurafenib and sabutoclax at lower doses, thereby mitigating the deleterious side effects associated with vemurafenib treatment (skin lesions, low grade squamous cell carcinomas). Hence, the combination of sabutoclax and vemurafenib may provide a better clinical option for many melanoma patients.

Based on its ability to regulate multiple key melanoma pathways, we anticipated that *miR-32* treatment would be effective at reducing tumor growth *in vivo*. Here we validate the efficacy of *miR-32* expression on inhibiting tumor growth, which was more effective than vemurafenib. Moreover, the combination *miR-32* and vemurafenib was more effective than either vemurafenib or *miR-32* treatment alone. In fact, the *miR-32*/vemurafenib combination worked as effectively in inhibiting tumor growth as the combination of sabutoclax and vemurafenib, likely because *miR-32* and sabutoclax are equally effective at inhibiting *MCL-1*. Accordingly, there was no added therapeutic benefit from combining *miR-32* with sabutoclax, which were equally effective individually at inhibiting *MCL-1*.

## Conclusions

Taken together, our results demonstrate that the loss of two tumor suppressors (*miR-32* and *ARF*) in concert with overexpression of oncogenic *MCL-1* represent key complementary drivers in melanomagenesis. The data also suggest that melanoma’s recalcitrance to many forms of therapy may lie, at least in part, in its ability to circumvent apoptosis by downregulating *ARF*/*miR-32* and/or upregulating *MCL-1*. We posit that *MCL-1* inhibition via *miR-32* or sabutoclax can be effective therapy, alone or in combination with other agents, in a variety of human melanoma subtypes that currently have no effective treatment options available in the clinic.

## Supporting Information

S1 Fig Gene expression analysis of the ARF-deficient (^-/-^ and ^-/+^) and INK4a-deficient (^-/-^ and ^-/+^) melanomas from the HGF/SF transgenic mice. A, Gene expression analysis identified MCL-1 as one of the top overexpressed genes associated with ARF loss. The significantly up and down regulated genes are listed. B, The principal component analysis is presented.
(PDF)

S2 Fig Gene network analysis of the top over-represented genes in tumors lacking ARF. Top over-represented gene networks were analyzed using ingenuity pathway analysis software identified MCL-1 linked to NFkB, interferon alpha, RNA polymerase II, follicle-stimulating hormone and cyclin D2 pathways. We hypothesize that there may be a direct regulation of MCL-1 through the CDKN2A locus by ARF (represented by a dotted red line).
(PDF)

S3 Fig The miR-32 chromosomal location. A, miR-32 is located on chromosome 9q31 (in the NR_029506.1 noncoding region) and is highly conserved between species (according to miRcode the miR-32 gene is conserved 89% among primates and 61% among mammals) B, miR-32 stem loop structure is presented.
(PDF)

S4 Fig MCL-1 is a strong candidate for an oncogene. A meta-analysis was used to show that compared to normal tissue MCL-1 is highly upregulated in 57 cancer data sets, whereas E2F-1, as a positive control, was upregulated in 45 cancer data sets. Actin was used as a negative control for this analysis and was upregulated in only 8 cancer types.
(PDF)

S5 Fig The Kaplan-Meir analysis and log-rank test were used to determine the association of drug treatment to survival. A375 melanoma cells were grown in athymic nude mice and treated as indicated. In this experiment no significant difference was observed between the control (C) and vemurafeninb-treated groups (V) in terms of overall survival, with a 50% survival of 60 days. Mice treated with Sabutoclax (S) had a 50% survival of 110 days. In contrast, mice treated with sabutoclax plus vemurafeninb (S+V) lived longer than one year. ***P-value < 0.0007, as determined by ANOVA (5-25nM). S+V-1 (V25nM+S25nM), S+V-2 (V12.5nM+S12.5nM), S+V-3 (V5nM+S5nM). Humane endpoints were used for the animal survival study and all efforts were made to minimize suffering (see methods for details).
(PDF)

S1 Table GO analysis of the microarray data. GO analysis of Ink4a^-/-, +/+^ (A) and ARF^-/-, +/+^ (B) melanomas revealed that loss of ARF is associated with more aggressive tumors and upregulation of DNA synthesis and repair machinery.
(PDF)

S2 Table An integrated analysis of microRNA-predicted sites in the 3’UTR of MCL-1 mRNA. The analysis was performed using GO miR software that integrates multiple target prediction algorithms (miRanda, TargetScan, PicTar4way, RNAhybrid, TraBase, PicTar5way).
(PDF)

S3 Table MCL-1 inhibitor sabutoclax is highly synergistic in combination with vemurafenib in melanoma cells. Synergistic effects of MCL-1 inhibitor sabutoclax with vemurafenib was tested using Chou-Talalay method using melanoma cell lines A375 (BRAFV600E/PTEN WT) and YUGEN8 (BRAFV600E/PTEN Null). Average IC-50 values from four different experiments are presented along with standard deviation. CI: combination index values.
(PDF)
