# Supplementary figures and images for: Integrated Genomics Identifies miR-32/MCL-1 Pathway as a Critical Driver of Melanomagenesis: Implications for miR-Replacement and Combination Therapy
Source: PLoS One. 2016 Nov 15;11(11):e0165102. doi: 10.1371/journal.pone.0165102 (PMC5113037; doi:10.1371/journal.pone.0165102)

A

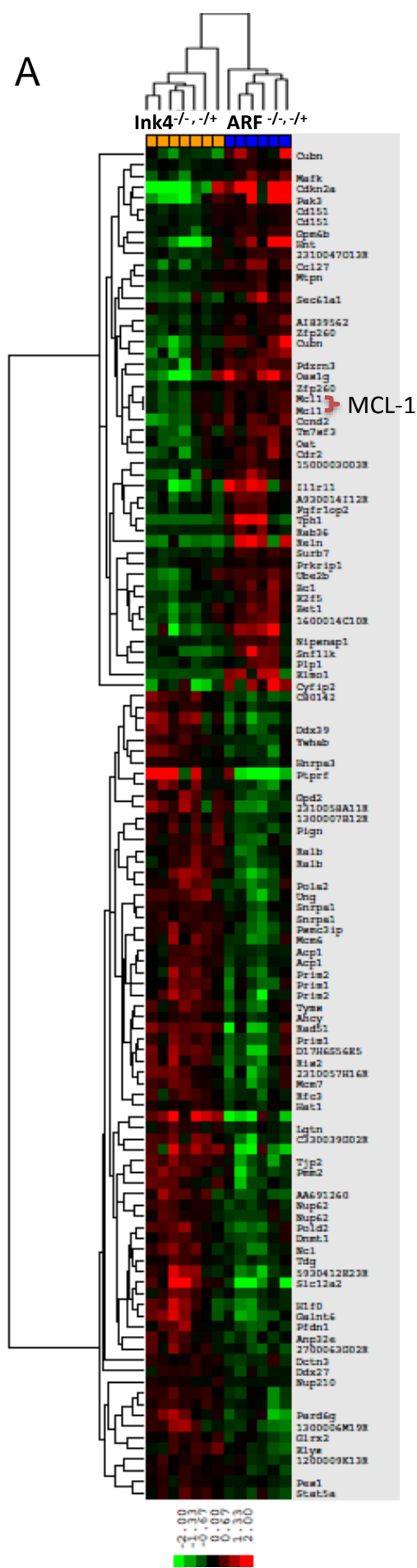

B

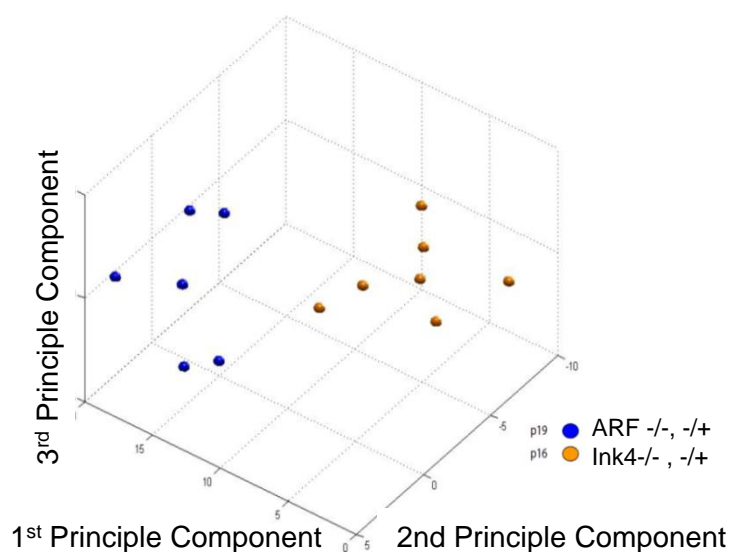

Supplement: S1 Fig — A, Gene expression analysis identified MCL-1 as one of the top overexpressed genes associated with ARF loss. The significantly up and down regulated genes are listed. B, The principal component analysis is presented. (PDF) [file pone.0165102.s001.pdf]

A  
Chr9  
(q31.3)

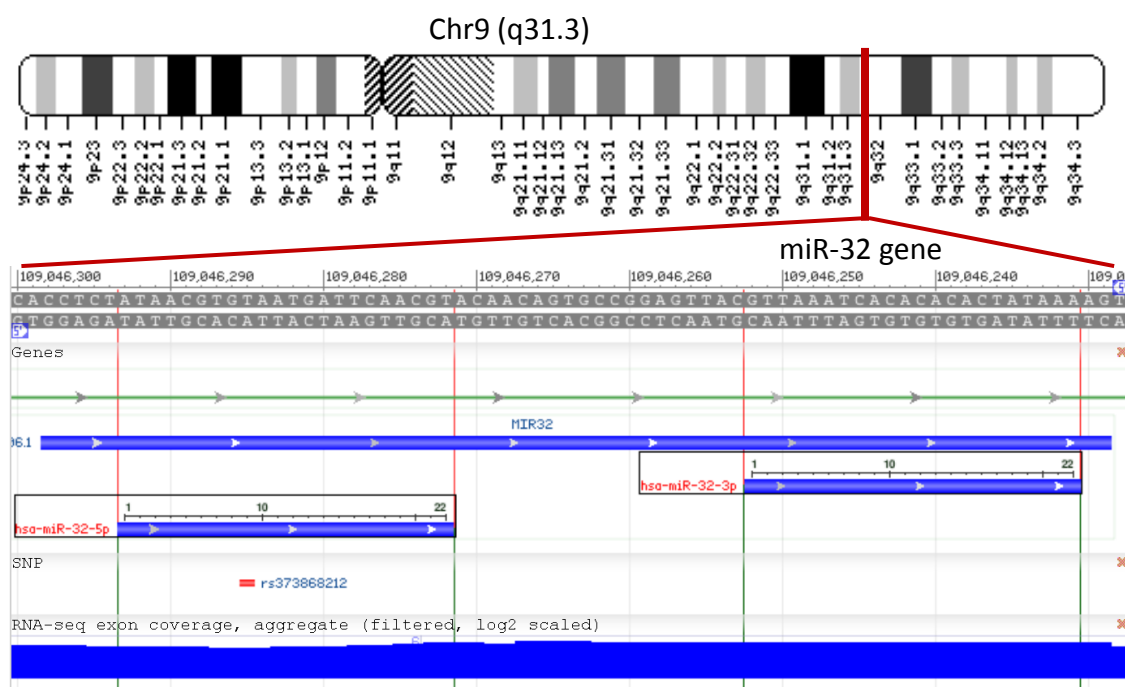

B

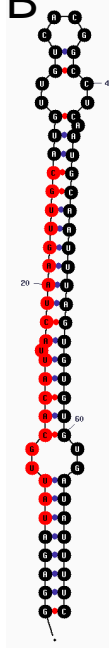

Supplement: S3 Fig — A, miR-32 is located on chromosome 9q31 (in the NR_029506.1 noncoding region) and is highly conserved between species (according to miRcode the miR-32 gene is conserved 89% among primates and 61% among mammals) B, miR-32 stem loop structure is presented. (PDF) [file pone.0165102.s003.pdf]

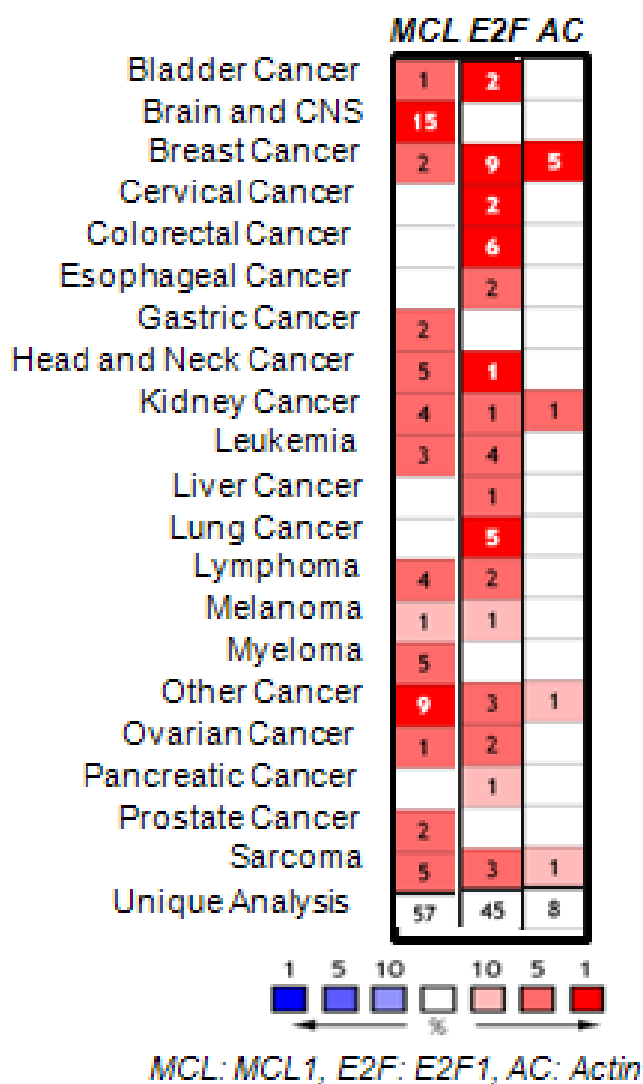

Supplement: S4 Fig — A meta-analysis was used to show that compared to normal tissue MCL-1 is highly upregulated in 57 cancer data sets, whereas E2F-1, as a positive control, was upregulated in 45 cancer data sets. Actin was used as a negative control for this analysis and was upregulated in only 8 cancer types. (PDF) [file pone.0165102.s004.pdf]

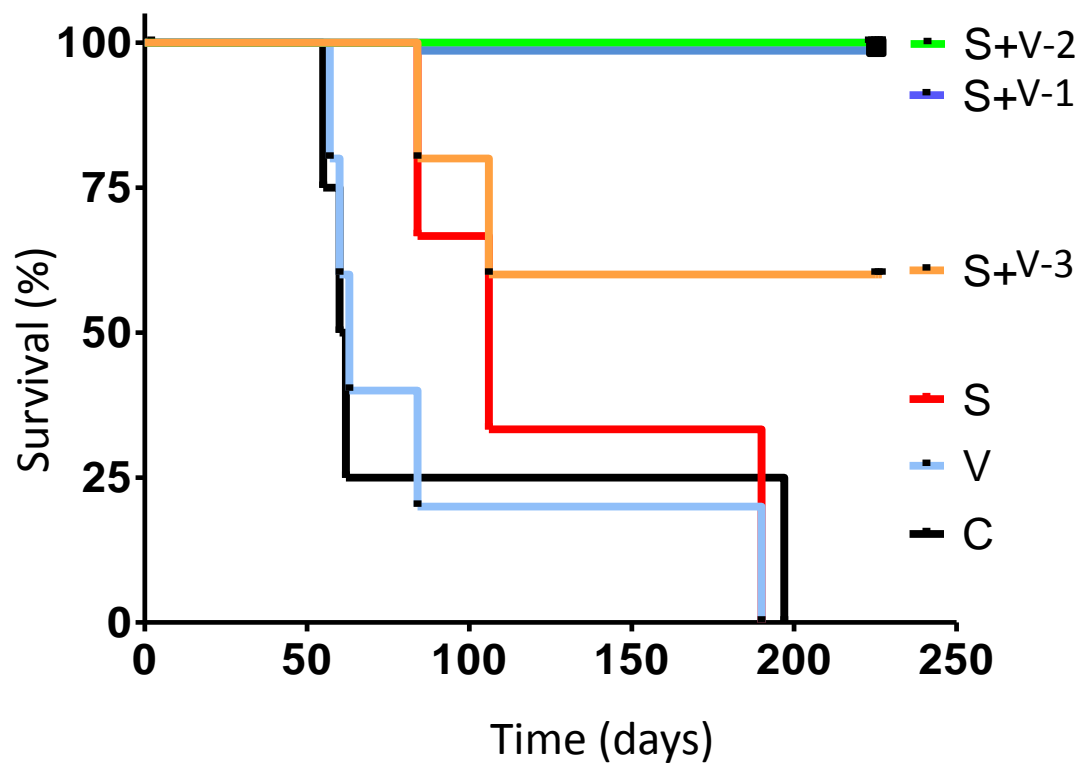

Supplement: S5 Fig — A375 melanoma cells were grown in athymic nude mice and treated as indicated. In this experiment no significant difference was observed between the control (C) and vemurafeninb-treated groups (V) in terms of overall survival, with a 50% survival of 60 days. Mice treated with Sabutoclax (S) had a 50% survival of 110 days. In contrast, mice treated with sabutoclax plus vemurafeninb (S+V) lived longer than one year. ***P-value < 0.0007, as determined by ANOVA (5-25nM). S+V-1 (V25nM+S25nM), S+V-2 (V12.5nM+S12.5nM), S+V-3 (V5nM+S5nM). Humane endpoints were used for the animal survival study and all efforts were made to minimize suffering (see methods for details). (PDF) [file pone.0165102.s005.pdf]
