# Supplementary material for: Integrated Genomics Identifies miR-32/MCL-1 Pathway as a Critical Driver of Melanomagenesis: Implications for miR-Replacement and Combination Therapy
Source: PLoS One. 2016 Nov 15;11(11):e0165102. doi: 10.1371/journal.pone.0165102 (PMC5113037; doi:10.1371/journal.pone.0165102)
Supplement: S1 Table — GO analysis of Ink4a-/-, +/+ (A) and ARF-/-, +/+ (B) melanomas revealed that loss of ARF is associated with more aggressive tumors and upregulation of DNA synthesis and repair machinery. (PDF) [file pone.0165102.s006.pdf]

S1 Table-A. Ink4A -/+ and -/- Melanoma GOs

Total	Change	P-Value	GO ID	Term
45	3	0.0005	19887	protein kinase regulator activity
47	3	0.0006	19207	kinase regulator activity
14	2	0.0011	6469	negative regulation of protein kinase activity
14	2	0.0011	43086	negative regulation of enzyme activity
15	2	0.0013	4860	protein kinase inhibitor activity
16	2	0.0015	19210	kinase inhibitor activity
23	2	0.003	82	G1/S transition of mitotic cell cycle
24	2	0.0033	48468	cell development
1	1	0.0036	46219	indolalkylamine biosynthesis
1	1	0.0036	6587	serotonin biosynthesis from tryptophan
1	1	0.0036	4587	ornithine-oxo-acid transaminase activity
1	1	0.0036	4510	tryptophan 5-monooxygenase activity
1	1	0.0036	42435	indole derivative biosynthesis
1	1	0.0036	42428	serotonin metabolism
1	1	0.0036	42427	serotonin biosynthesis
1	1	0.0036	42326	negative regulation of phosphorylation
1	1	0.0036	30170	pyridoxal phosphate binding
1	1	0.0036	45936	negative regulation of phosphate metabolism
2	1	0.0072	7529	establishment of synaptic specificity at neuromuscular junction
38	2	0.0082	45859	regulation of protein kinase activity
1855	13	0.0095	5515	protein binding

S1 Table-B. ARF -/+ and -/- Melanoma GOs: DNA synthetic machinery upregulated

Total	Change	P-Value	GO ID	Term
2	2	0	6273	lagging strand elongation
2	2	0	6269	DNA replication, synthesis of RNA primer
25	4	0	6261	DNA-dependent DNA replication
70	7	0	6260	DNA replication
295	14	0	6259	DNA metabolism
10	3	0	30894	replisome
449	12	0	7049	cell cycle
141	7	0	5694	chromosome
14	3	0	5657	replication fork
2070	26	0	5634	nucleus
89	7	0	67	DNA replication and chromosome cycle
2	2	0	3896	DNA primase activity
592	12	0	8283	cell proliferation
3	2	0.0001	6271	DNA strand elongation
3	2	0.0001	5658	alpha DNA polymerase:primase complex
18	3	0.0001	8094	DNA-dependent ATPase activity
3716	32	0.0003	43229	intracellular organelle
3716	32	0.0003	43226	organelle
4341	35	0.0005	5622	intracellular
9	2	0.0009	6270	DNA replication initiation
9	2	0.0009	4727	prenylated protein tyrosine phosphatase activity
3284	28	0.0015	43231	intracellular membrane-bound organelle
3284	28	0.0015	43227	membrane-bound organelle
1684	18	0.0015	3676	nucleic acid binding
1709	18	0.0018	6139	nucleobase, nucleoside, nucleotide and nucleic acid metabolism
179	5	0.0024	42623	ATPase activity, coupled
187	5	0.0029	16887	ATPase activity
16	2	0.0031	46165	alcohol biosynthesis
16	2	0.0031	19319	hexose biosynthesis
16	2	0.0031	46364	monosaccharide biosynthesis
280	6	0.0033	3723	RNA binding
285	6	0.0036	17111	nucleoside-triphosphatase activity
301	6	0.0047	16462	pyrophosphatase activity
20	2	0.0048	6306	DNA methylation
20	2	0.0048	6305	DNA alkylation
304	6	0.0049	16818	hydrolase activity, acting on acid anhydrides, in phosphorus-containing anhydrides
304	6	0.0049	16817	hydrolase activity, acting on acid anhydrides
1	1	0.0052	46073	dTMP metabolism
1	1	0.0052	6231	dTMP biosynthesis
1	1	0.0052	4844	uracil DNA N-glycosylase activity
1	1	0.0052	4799	thymidylate synthase activity
1	1	0.0052	4368	glycerol-3-phosphate dehydrogenase activity
1	1	0.0052	9177	pyrimidine deoxyribonucleoside monophosphate biosynthesis
1	1	0.0052	9176	pyrimidine deoxyribonucleoside monophosphate metabolism
1	1	0.0052	9162	deoxyribonucleoside monophosphate metabolism
1	1	0.0052	9157	deoxyribonucleoside monophosphate biosynthesis
1	1	0.0052	9130	pyrimidine nucleoside monophosphate biosynthesis
1	1	0.0052	9129	pyrimidine nucleoside monophosphate metabolism
1	1	0.0052	42083	5,10-methylenetetrahydrofolate-dependent methyltransferase activity
1	1	0.0052	19510	S-adenosylhomocysteine catabolism
1	1	0.0052	19031	viral envelope
1	1	0.0052	17056	structural constituent of nuclear pore
1	1	0.0052	46498	S-adenosylhomocysteine metabolism
21	2	0.0053	5643	nuclear pore
21	2	0.0053	46930	pore complex
68	3	0.0054	16779	nucleotidyltransferase activity
22	2	0.0058	793	condensed chromosome
24	2	0.0069	6304	DNA modification
24	2	0.0069	3899	DNA-directed RNA polymerase activity
335	6	0.0078	46907	intracellular transport
2451	21	0.0089	8151	cell growth and/or maintenance
695	9	0.0093	43232	intracellular non-membrane-bound organelle
695	9	0.0093	43228	non-membrane-bound organelle
