# Supplementary material for: Integrated Genomics Identifies miR-32/MCL-1 Pathway as a Critical Driver of Melanomagenesis: Implications for miR-Replacement and Combination Therapy
Source: PLoS One. 2016 Nov 15;11(11):e0165102. doi: 10.1371/journal.pone.0165102 (PMC5113037; doi:10.1371/journal.pone.0165102)
Supplement: S2 Table — The analysis was performed using GO miR software that integrates multiple target prediction algorithms (miRanda, TargetScan, PicTar4way, RNAhybrid, TraBase, PicTar5way). (PDF) [file pone.0165102.s007.pdf]

S2 Table. An integrated analysis of microRNA-predicted sites in the 3'UTR of MCL-1 mRNA. The analysis was performed using GO miR software that integrates multiple target prediction algorithms (miRanda, TargetScan, PicTar4way, RNAhybrid, TraBase, PicTar5way).

microRNA	Comple-mentarity	Conservation	Regulates short/long forms of MCL-1 mRNA
miR-25/32/92/92ab/363/367	8mer	Highly conserved	All forms
miR-193ab	8mer	Conserved	All forms
miR-153	7mer-8	Conserved	All forms
miR-29ac	8mer	Conserved	Long form
miR-125/351	8mer	Conserved	Long form
miR-17-5p/20/93.mr/106/519.d	8mer	Conserved	Long form
miR-181	7mer	Conserved	Long form
miR-27ab	8mer	Poorly conserved	Long form
miR-15/16/195/424/497	7mer	Poorly conserved	Long form
miR-135	7mer	Poorly conserved	Long form
miR-133	7mer	Poorly conserved	Long form
miR-106/302	7mer	Non conserved	Long form
