# Supplementary material for: Integrated Genomics Identifies miR-32/MCL-1 Pathway as a Critical Driver of Melanomagenesis: Implications for miR-Replacement and Combination Therapy
Source: PLoS One. 2016 Nov 15;11(11):e0165102. doi: 10.1371/journal.pone.0165102 (PMC5113037; doi:10.1371/journal.pone.0165102)
Supplement: S3 Table — Synergistic effects of MCL-1 inhibitor sabutoclax with vemurafenib was tested using Chou-Talalay method using melanoma cell lines A375 (BRAFV600E/PTEN WT) and YUGEN8 (BRAFV600E/PTEN Null). Average IC-50 values from four different experiments are presented along with standard deviation. CI: combination index values. (PDF) [file pone.0165102.s008.pdf]

S3 Table. MCL-1 inhibitor sabutoclax is highly synergistic in combination with vemurafenib in melanoma cells. Synergistic effects of MCL-1 inhibitor sabutoclax with vemurafenib was tested using Chou-Talalay method using melanoma cell lines A375 (BRAFV600E/PTEN WT) and YUGEN8 (BRAFV600E/PTEN Null). Average IC-50 values from four different experiments are presented along with standard deviation. CI: combination index values

A375 cells INK4A/ARF-/-, BRAFV600E, PTENwt			YUGEN8 cells INK4A/ARF-/-, BRAFV600E, PTENnull		
Sabutoclax nM	Vemurafenib nM	CI	Sabutoclax nM	Vemurafenib nM	CI
0.5	0.5	0.0340	0.5	0.5	0.0180
1	1	0.0680	1	1	0.0320
2	2	0.0350	2	2	0.0440
3.9	3.9	0.0750	3.9	3.9	0.0970
7.8	7.8	0.0290	7.8	7.8	0.0300
15.6	15.6	0.0590	15.6	15.6	0.0590
31.25	31.25	0.1170	31.25	31.25	0.1190
62.5	62.5	0.2350	62.5	62.5	0.2380
125	125	0.3520	125	125	0.3450
250	250	0.7040	250	250	0.6900

CI<1 Highly Synergistic
CI=1 Additive
CI>1 Antagonistic
