# Supplementary material for: Integrated Genomics Identifies miR-32/MCL-1 Pathway as a Critical Driver of Melanomagenesis: Implications for miR-Replacement and Combination Therapy
Source: PLoS One. 2016 Nov 15;11(11):e0165102. doi: 10.1371/journal.pone.0165102 (PMC5113037; doi:10.1371/journal.pone.0165102)
Supplement: S2 Fig — Top over-represented gene networks were analyzed using ingenuity pathway analysis software identified MCL-1 linked to NFkB, interferon alpha, RNA polymerase II, follicle-stimulating hormone and cyclin D2 pathways. We hypothesize that there may be a direct regulation of MCL-1 through the CDKN2A locus by ARF (represented by a dotted red line). (PDF) [file pone.0165102.s002.pdf]

S2 Fig

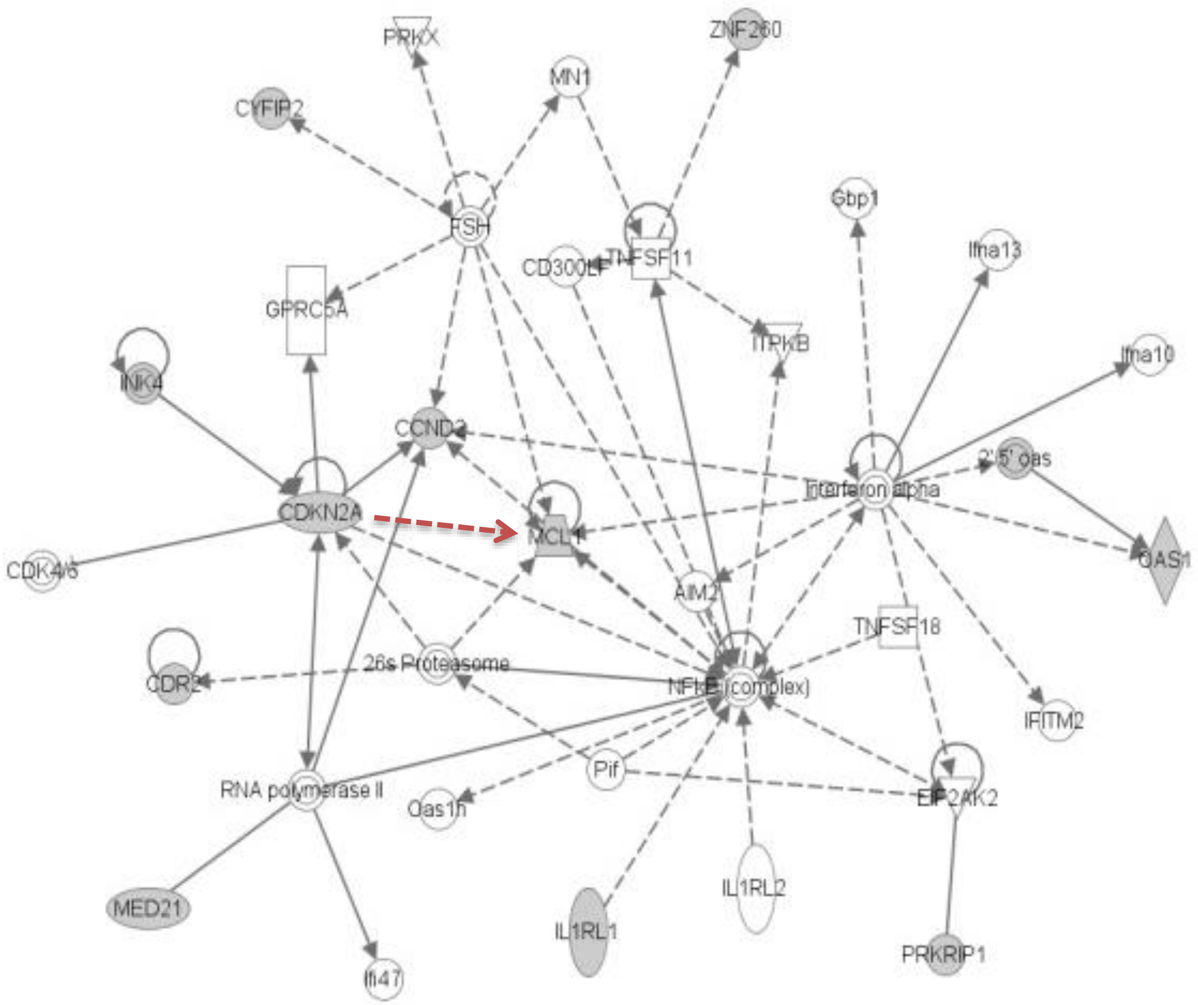

PRKX
ZNF260
MN1
CYFIP2
FSH
Gbp1
CD300LF
TNFSF11
Ifna13
GPRC5A
ITPKB
Ifna10
INK4
CCND2
2'5' oas
Interferon alpha
CDKN2A
MCL1
OAS1
CDK4/6
AIM2
TNFSF18
CDR2
26s Proteasome
NFkB (complex)
IFITM2
Pif
RNA polymerase II
Oas1h
EIF2AK2
MED21
IL1RL2
IL1RL1
PRKRIP1
Ifi47
